# Improved therapeutic effects on vascular intimal hyperplasia by mesenchymal stem cells expressing MIR155HG that function as a ceRNA for microRNA‐205

**DOI:** 10.1111/jcmm.18351

**Published:** 2024-05-02

**Authors:** Xiao Bai, Zaiwen Qi, Chuanliang Cai, Hao Song, Guangmin Song, Xin Zhao

**Affiliations:** ^1^ Department of Cardiovascular Surgery Qilu Hospital of Shandong University Jinan China; ^2^ Thoracoscopy Institute of Cardiac Surgery Shandong University Jinan China; ^3^ The Fifth People's Hospital of Jinan Jinan China

**Keywords:** ceRNA, intimal hyperplasia, mesenchymal stem cells, microRNA‐205, MIR155HG

## Abstract

Coronary artery bypass grafting (CABG) is an effective treatment for coronary heart disease, with vascular transplantation as the key procedure. Intimal hyperplasia (IH) gradually leads to vascular stenosis, seriously affecting the curative effect of CABG. Mesenchymal stem cells (MSCs) were used to alleviate IH, but the effect was not satisfactory. This work aimed to investigate whether lncRNA MIR155HG could improve the efficacy of MSCs in the treatment of IH and to elucidate the role of the competing endogenous RNA (ceRNA). The effect of MIR155HG on MSCs function was investigated, while the proteins involved were assessed. IH was detected by HE and Van Gieson staining. miRNAs as the target of lncRNA were selected by bioinformatics analysis. qRT‐PCR and dual‐luciferase reporter assay were performed to verify the binding sites of lncRNA‐miRNA. The apoptosis, Elisa and tube formation assay revealed the effect of ceRNA on the endothelial protection of MIR155HG‐MSCs. We observed that MIR155HG improved the effect of MSCs on IH by promoting viability and migration. MIR155HG worked as a sponge for miR‐205. MIR155HG/miR‐205 significantly improved the function of MSCs, avoiding apoptosis and inducing angiogenesis. The improved therapeutic effects of MSCs on IH might be due to the ceRNA role of MIR155HG/miR‐205.

−

## 1 INTRODUCTION

Coronary heart disease accounts for nearly 7 million deaths each year worldwide, with coronary artery bypass grafting (CABG) surgery being the effective treatment in serious cases.^1^ The bypass restores the blood flow to the ischemic myocardium, consequently improving myocardial function and relieving ischemic symptoms.^2^ Saphenous vein grafts are the most frequently used vessels in CABG surgery, but 10%–25% experience an occlusion within the first year after CABG.^3^, ^4^ The intimal hyperplasia (IH) of the grafted vessels inevitably and gradually occurs.^5^

The main cellular components of the vascular wall are vascular endothelial cells (VECs) and vascular smooth muscle cells (VSMCs).^6^, ^7^ The pathological process of IH starts with the injury of VECs. VECs apoptosis is inevitable due to surgical procedures, ischemia–reperfusion, luminal pressure and changes in blood flow direction. This induces inflammation and stimulates the transformation of the contractile phenotype of VSMCs into the synthetic phenotype.^8^, ^9^ In addition, the two types of cells also “cross‐talk” with each other through extracellular vesicles under pathological conditions.^10^ These combined effects lead to IH, lumen stenosis and even occlusion.^11^, ^12^ MSCs are widely used for tissue repair. A study confirms that homing MSCs participate in endothelial repair and inhibit IH through paracrine.^13^ Another study reports that the repressor/activator protein‐1 (RAP1), also called Terf2ip, is essential to maintain the immunomodulatory and paracrine function of MSCs.^14^

Long noncoding RNA (lncRNA) is a non‐protein coding RNA molecule with a length of 200–100,000 nt mainly involved in transcription or post‐transcriptional regulation, protein transport and mRNA degradation.^15^, ^16^ The miR‐155 host gene (MIR155HG) (NCBI Reference Sequence: NR_132107.1) is a lncRNA involved in apoptosis, inflammation and tumorigenesis.^17^, ^18^ It has been reported that MIR155HG promotes the migration and invasion of gastric cancer cells.^19^ Thus, the effect of MIR155HG on the activity of MSCs and its promoting effect on IH therapy were assessed in this work.

LncRNAs act as competing endogenous RNAs (ceRNAs), competitively sponging microRNAs (miRNA) to protect other endogenous RNAs inhibited by miRNA.^20^ Therefore, the lncRNA‐miRNA ceRNA mechanism could play a powerful gene regulation role. MALAT1 is a ceRNA for miR‐205, and decreased miR‐205 expression resulted in the upregulation of vascular endothelium growth factor (VEGF) production and improved in vitro endothelial cell tube formation.^21^ As a tumour suppressor, the overexpression of miR‐205 reduces renal cell carcinoma cell proliferation, invasion and migration.^22^

Hence, this study aimed to investigate the functional improvement of MIR155HG on MSCs and explain the role and mechanism of MIR155HG/miR‐205 ceRNA. In this way, MSCs regulated by ceRNA can be used to treat IH more effectively.

## 2 MATERIALS AND METHODS

### 2.1 MSC culture, identification and viral transfection

MSCs were obtained from Cyagen Biosciences Inc. (Shanghai, China) and cultured according to the manufacturer's guidelines. The cells were grown in DMEM (Gibco, USA) for 48 h at 37°C and 5% CO_2_. The medium was changed every 2 days. The NF‐κB inhibitor BMS‐345541 was purchased from MCE (New Jersey, USA). MSCs were treated with 5 μM BMS‐345541 for 2 h.

MSCs were identified by flow cytometry using stem cell surface markers according to the manufacturer's protocol. Antibodies against the MSC markers, anti‐CD29, anti‐CD34, anti‐CD45 and anti‐CD90, were purchased from BD Biosciences.

As regards functional assay, MSCs were transfected with MIR155HG‐expressing lentivirus vector (OLIGOBIO, Beijing, China) at an MOI of 60, or with miR‐205 mimic (GeneChem, China) at a concentration of 50 nM. Oligonucleotide transfection was performed using Lipofectamine 2000 reagent (Invitrogen, USA) according to the manufacturer's protocol.

### 2.2 Quantitative real‐time PCR (qRT‐PCR)

Total RNA was extracted using TRIzol reagent (Invitrogen, USA). Complementary DNA preparation and qRT‐PCR were routinely performed using PrimeScript RT reagent kit (Takara, Japan). Reverse transcription primers were designed and synthesized by GENEWIZ (Suzhou, China). Data were collected and analysed using the 2^−ΔΔCt^ method. Gene expression was first normalized against GAPDH or U6, and then compared to the experimental controls. The primer sequences: MIR155HG forward 5′‐GCTTGCTGAAGGCTGTATGC‐3′, MIR155HG reverse 5′‐GTCTTGTCA TCCTCCCACGG‐3′; RAP1 forward 5′‐GGAGAGGCAGACAACAAGCT‐3′, RAP1 reverse 5′‐TCCTCGTCTGGCTGTGTTTC‐3′; GAPDH forward 5′‐GATTTGGCC GTATCGGAC‐3′, GAPDH reverse 5′‐GAAGACGCCAGTAGACTC‐3′.

### 2.3 Western blot

Total proteins were obtained using RIPA lysis buffer. Protein concentration was detected using the BCA protein quantification kit. Protein samples were used for 10% SDS‐PAGE electrophoresis. Then, the proteins were transferred to a PVDF membrane, which was blocked with 5% BSA. The membrane was incubated at 4°C overnight with primary antibodies: anti‐p‐NF‐κB P65, anti‐NF‐κB P65 (ABclonal, USA); anti‐GAPDH (Proteintech, USA). Subsequently, the HRP Goat Anti‐Rabbit/Mouse IgG (H + L) (ABclonal, USA) was added. Immunoreactive proteins were visualized using the ECL kit (Amersham, USA).

### 2.4 Cell viability, cell cycle and apoptosis

Cell viability was detected using a CCK‐8 kit (MCE, New Jersey, USA). Cells were seeded onto 96‐well plates and incubated for 24–48 h. CCK‐8 10% solution was added to each well and incubated for 2 h. The absorbance was read at 450 nm using an enzyme micro‐plate reader. Cell cycle was detected using the Cycle test Plus DNA Reagent Kit (BD Biosciences, San Jose, CA, USA) according to the manufacturer's instruction.

In the apoptosis experiment, cells were treated with 200 μM hydrogen peroxide (H_2_O_2_) for 12 h to simulate the oxidative stress. Apoptosis of MSCs was measured by flow cytometry using Annexin V/PI apoptosis detection kit (Keygen, Nanjing, China).

### 2.5 Transwell migration

Cell migration assay was performed using transwell inserts (Corning, USA) with a filter of 8 μm pore. Approximately 5 × 10^4^ MSCs were seeded in the upper chamber, and 600 μL conditional medium was added to the lower chamber. Cells on the upper side of the membrane were wiped off after 24 h incubation. Then, cells on the lower surface were stained with 0.1% crystal violet. Images of five random fields were taken and cells were counted.

### 2.6 HE and Van Gieson staining

The external jugular vein was removed from the SD rat and connected to the infrarenal abdominal aorta in the same rat using the cuff technique.^13^ MSCs (1 × 10^6^) were injected into the caudal vein 24 h after surgery. Rats were humanely sacrificed 4 weeks later. All the experimental procedures were performed following the Institutional Animal Care and Use Committee guidelines.

Vein grafts were fixed in 4% paraformaldehyde and then embedded in paraffin. The paraffin specimens were cut into 4‐μm‐thick slices. Paraffin sections were dewaxed, then haematoxylin–eosin (HE) staining and Van Gieson staining were performed to assess IH according to the manufacturer's instruction.

### 2.7 Microarray analysis and prediction of binding sites

The microarray was used to detect the differential expression of miRNAs. The RNAhybrid and miRanda software were selected for target gene prediction, with the intersection obtained by the two methods as the result. The hierarchical clustering analysis of TPM (tags per million) values, miRNA Volcano Plot analysis and miRNA target gene GO function classification statistics were conducted to analyse miRNA expression in MSCs with MIR155HG transfection. In addition, we used the miRanda tool to conduct the binding analysis of MIR155HG and 10 miRNA sequences in order to obtain the potential binding sites of miRNA‐lncRNA.

### 2.8 Dual‐luciferase reporter assay

The wild‐type and mutant plasmids of the double luciferase reporter gene of MIR155HG, miR‐205 mimic and its negative control plasmids were synthesized by Shanghai GeneChem, Co., Ltd. Plasmids were transfected into 293 T cells using Lipofectamine 2000 reagent (Invitrogen, USA) and incubated for 24 h. Firefly and renal luciferase activities were continuously measured by a dual luciferase reporter gene assay kit (Promega, USA).

### 2.9 Enzyme‐linked immunosorbent assay (Elisa) assay

The conditional medium of cultured MSCs was collected and centrifuged at 1000 *g* for 20 min and the supernatant was collected. VEGF concentration in the supernatant was detected by an Elisa kit (ExCellBio, Shanghai, China) according to the manufacturer's instruction.

### 2.10 Endothelial cells tube formation

HUVECs were purchased from FuHeng Biology (Shanghai, China) and were cultured in ECM (ScienCell, USA) containing 5% fetal bovine serum and 1% endothelial cell growth supplements. Then HUVECs were collected and seeded at a density of 2 × 10^4^ per well on growth factor‐depleted Matrigel (BD Biosciences, USA) in 48‐well plates. Conditional medium collected from MSCs was added, and the tube formation was observed at 12 h. Microscopic fields containing tube‐like structures formed in the gel were photographed. The results were analysed by Image J software.

### 2.11 Statistical analysis

Statistical analysis was performed using SPSS version 20.0 software (SPSS, Chicago, USA). Experiments were carried out at least three times, and the results were presented as mean ± standard deviation. The two‐sample student *t*‐test or one‐way ANOVA was used to analyse the difference. A value of *p* < 0.05 was considered statistically significant.

## 3 RESULTS

### 3.1 Viral transfection of lncRNA MIR155HG in MSCs

**FIGURE 1:**
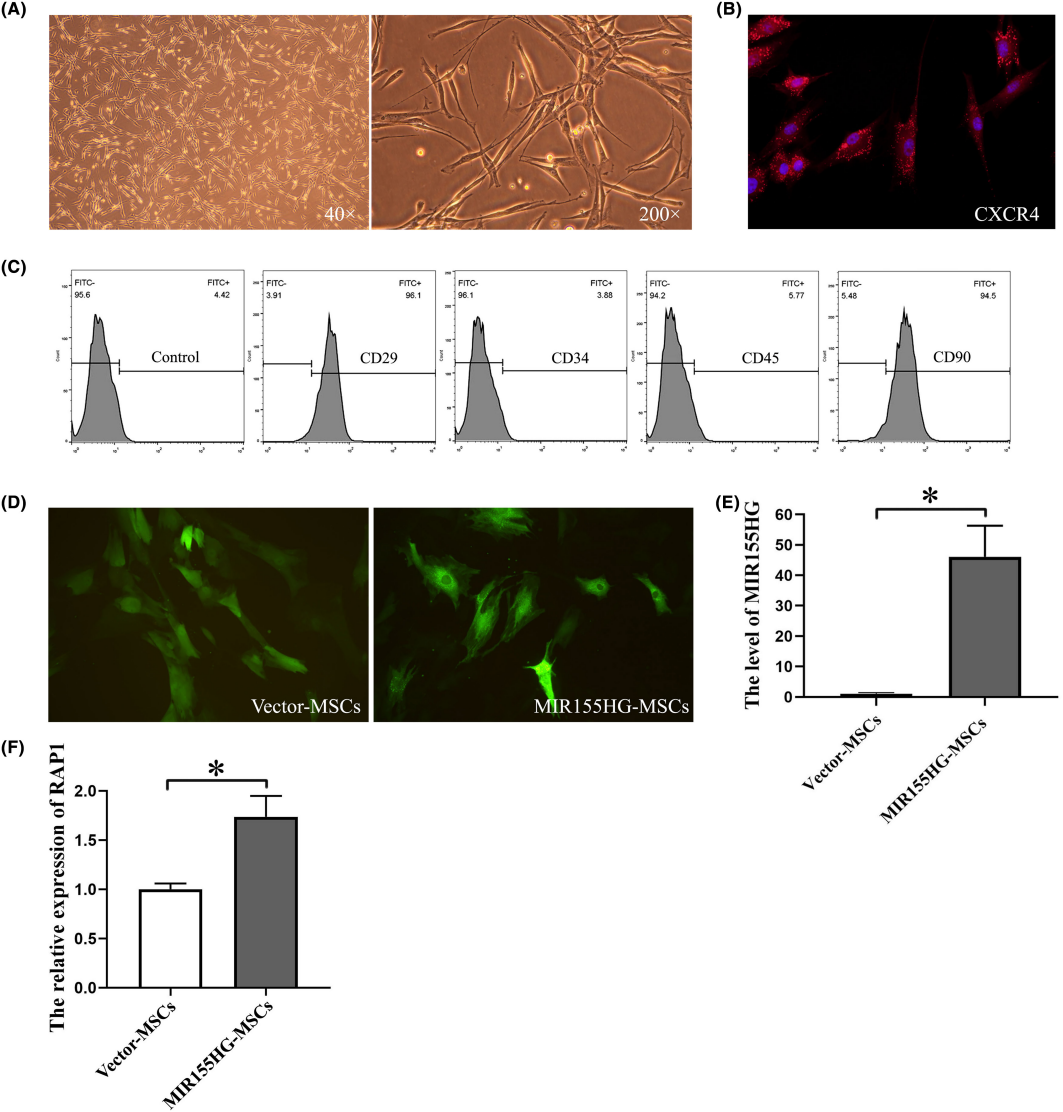
MSCs and MIR155HG transfection. (A) Microscopic view of fibroblast‐like MSCs. (B) CXCR4 (red) and DAPI (purple) immunostaining on MSCs. (C) Surface makers of MSCs by flow cytometric analysis. Cells were incubated with CD29, CD34, CD45 and CD90 antibodies. (D) Green fluorescence of MSCs transfected with lentivirus observed under fluorescence microscope. (E) Transfection efficiency of MIR155HG in MSCs verified by qRT‐PCR, *p < 0.05. (F) The expression of RAP1 in MSCs, *p < 0.05.

The cultured MSCs grew adhering to the bottom and had a fibroblast‐like morphology (Figure 1A). CXCR4 was expressed in MSCs (Figure 1B), which is a key molecule in the migration of MSCs. The cell surface markers CD29 and CD90 were expressed in MSCs, while CD34 and CD45 were not (Figure 1C). The cells used in this experiment were identified as MSCs. MSCs emitted strong green fluorescence after transfecting by GFP‐labelled lentiviral vector (Figure 1D). The expression of MIR155HG in the MIR155HG‐MSCs after viral transfection was significantly increased compared with that in the vector‐MSCs group (Figure 1E). The overexpression of MIR155HG in MSCs was successfully achieved. Moreover, the level of RAP1 in MSCs was up‐regulated by MIR155HG overexpression (Figure 1F). It suggests that MIR155HG‐MSCs may have better paracrine function.

### 3.2 MIR155HG promotes the proliferation and migration of MSCs through NF‐κB pathway

**FIGURE 2:**
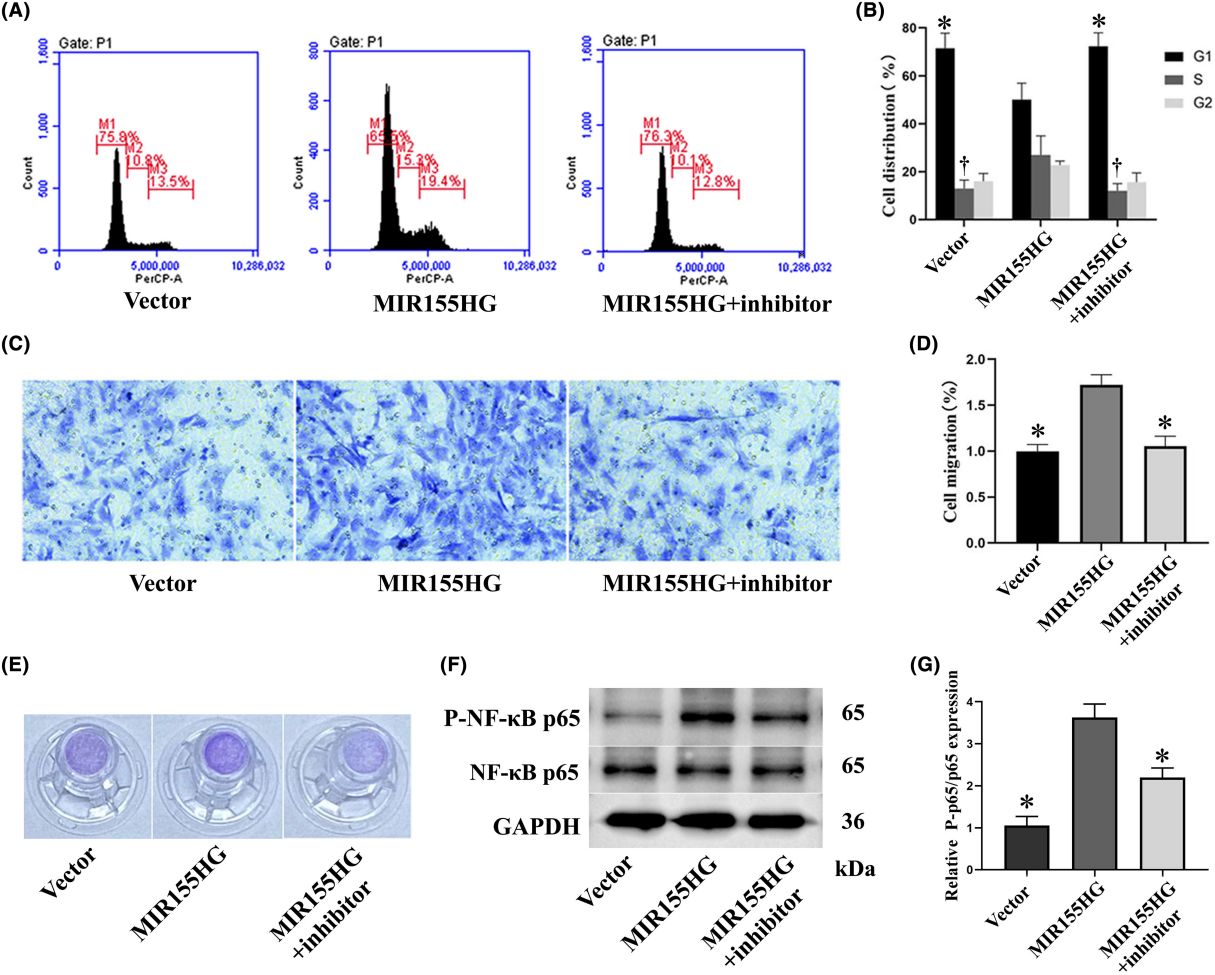
Effects of MIR155HG and NF‐κB pathway on proliferation and migration of MSCs. (A) Representative images of flow cytometry. (B) Cell distribution in different phase of the cell cycles determined by flow cytometry, *p < 0.05 or ^†^ *p* < 0.05 vs. MIR155HG group. (C) Representative images of transwell assay. (D) Quantitative analysis of cell migration, *p < 0.05 vs. MIR155HG group. (E) Images of the transwell chambers used in the experiment. (F) Representative western blots of p‐NF‐κB p65/ NF‐κB p65 expression in MSCs. (G) Quantitative analysis of western blots, *p < 0.05 vs. MIR155HG group.

MIR155HG significantly reduced the proportion of cells in G1 phase. The NF‐κB inhibitor BMS‐345541 reversed the decrease in the proportion of G1 phase cells. In addition, MIR155HG increased the proportion of cells in S phase, but the NF‐κB inhibitor significantly reversed this effect (Figure 2A,B). The results showed that MIR155HG promoted the proliferation of MSCs, and this effect was achieved by the NF‐κB pathway. Moreover, the effect of MIR155HG on the migration of MSCs revealed that the number of migratory cells in the MIR155HG group was significantly increased than in the vector group. NF‐κB inhibitor inhibited the improvement of cell migration (Figure 2C–E). The expression of phosphorylated NF‐κB p65 in the MIR155HG group was significantly increased than in the vector group. MIR1555HG effectively activated the NF‐κB pathway (Figure 2F,G). These results revealed that MIR155HG promoted the proliferation and migration of MSCs through the NF‐κB pathway.

### 3.3 MIR155HG‐MSCs significantly attenuates vein graft IH

**FIGURE 3:**
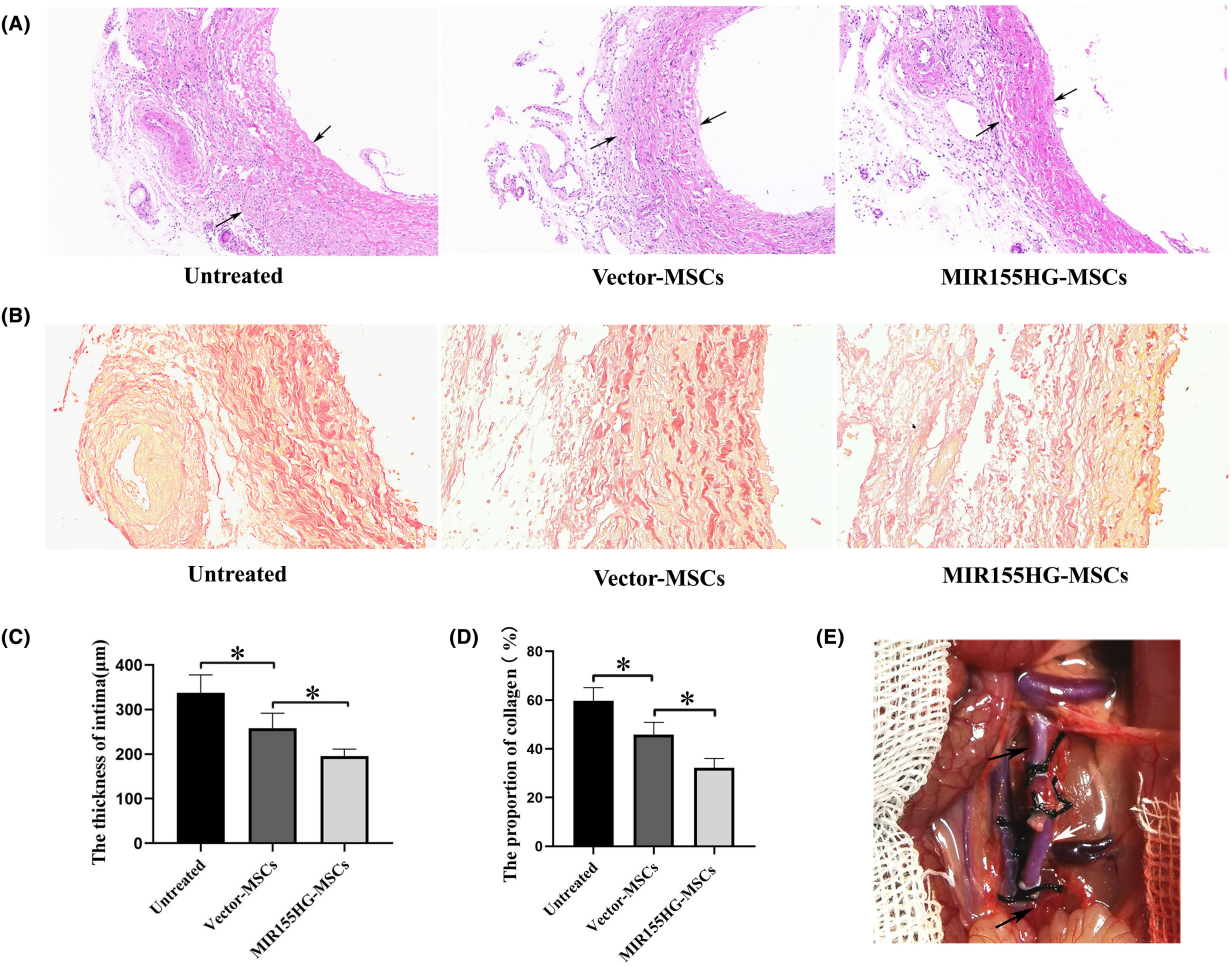
Role of MIR155HG in the treatment of IH by MSCs. (A) Representative HE staining images (×15) of vein graft. The black arrow indicates the intima of the vein graft. (B) Representative Van Gieson staining images (×30) of vein graft. The collagen is stained red and the muscle is stained yellow. (C, D) Quantitative analysis of A and B, *p < 0.05. (E) Rat model of autogenous vein transplantation. The white arrow indicates the grafted vein, the black arrow indicates the abdominal aorta.

The vascular intima of the vector‐MSCs group was thinner than that of the untreated group. And the IH was significantly alleviated in the MIR155HG‐MSCs group compared with the vector‐MSCs group (Figure 3A). In addition, the collagen deposition of the vein graft was also reduced in the MIR155HG‐MSCs group (Figure 3B). It was confirmed that MSCs regulated by MIR155HG more effectively delayed the progression of vein graft IH (Figure 3C,D). The model of autologous external jugular vena‐abdominal aorta transplantation simulated the pathological process of IH and the gradual narrowing of the lumen after CABG surgery in clinical practice (Figure 3E).

### 3.4 LncRNA MIR155HG functions as a ceRNA for miR‐205

**FIGURE 4:**
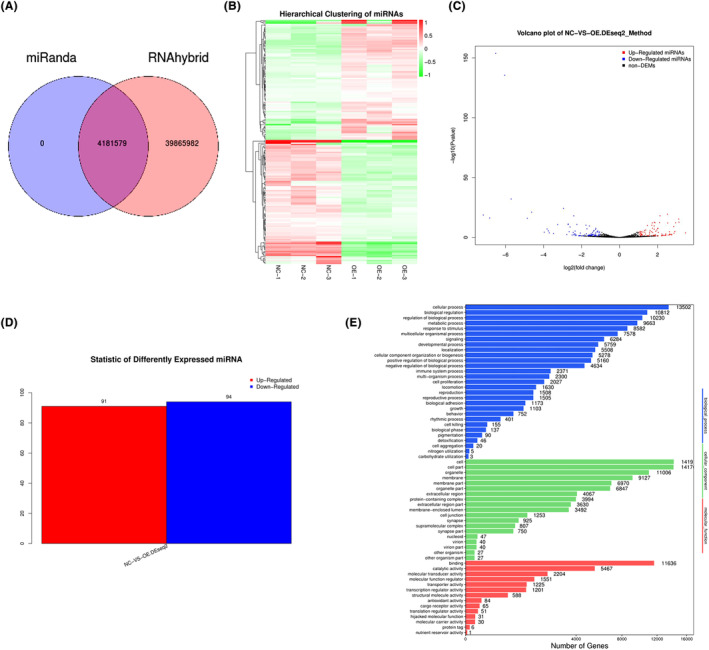
Sequencing analysis of differentially expressed MIR155HG‐related miRNAs. (A) Venn diagram of predicted results of the targeted genetic locus. (B) Clustering heat map of differentially expressed miRNA. The clustering of miRNAs with similar expression patterns into classes was used to predict the functions of unknown miRNAs or new functions of known miRNAs. (C) Volcano maps of differentially expressed miRNA. The volcano map was used to evaluate the differences in miRNA expression in the two different samples, as well as the statistical significance of the differences. (D) Differentially expressed miRNA. (E) GO functional classification map of differentially expressed miRNA.

The result of miRNA target gene prediction is shown in Figure 4A. A large difference in miRNA expression between two groups was found. A total of 91 miRNAs were significantly up‐regulated, while 94 miRNAs were down‐regulated (Figure 4B–D). The differential expression of miRNA in different samples might be related to the functional changes of some genes (Figure 4E).

**FIGURE 5:**
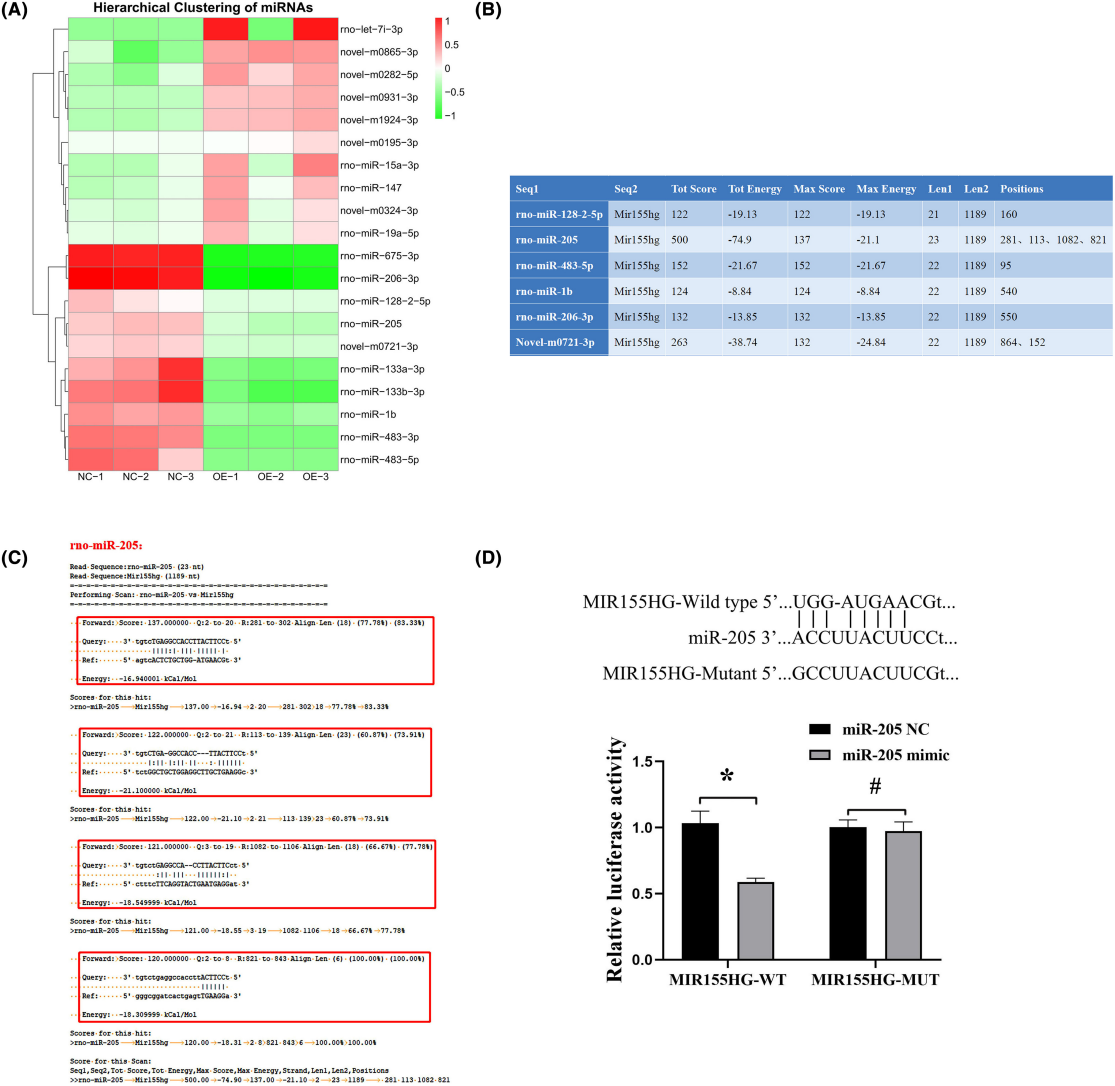
MIR155HG functions as a ceRNA for miR‐205. (A) Clustering heat map of the top 10 significantly up‐regulated and down‐regulated miRNAs. (B) Results of the combined analysis of miRNAs and MIR155HG by miRanda software according to threshold values. (C) Specific binding sequences of MIR155HG to miR‐205. (D) Dual‐luciferase reporter assay was used to detect the binding sites between MIR155HG and miR‐205, *p < 0.05; ^#^ *p* > 0.05.

The top 10 significantly down‐regulated miRNAs were selected and the binding analysis of MIR155HG and 10 miRNA sequences revealed the potential binding sites of miRNA and lncRNA, as shown in Figure 5A. The higher the score value and the lower the energy value in the table, the more stable the binding between miRNA and MIR155HG, and the higher the probability that the miRNA is the target gene of MIR155HG (Figure 5B). miR‐205 was selected for further study after binding sites analysis and literature search. Luciferase assays also showed that miR‐205 could specifically combine with MIR155HG (Figure 5C,D). These results confirmed that MIR155HG worked as a ceRNA for miR‐205.

### 3.5 miR‐205 suppresses the viability and migration of MSCs

**FIGURE 6:**
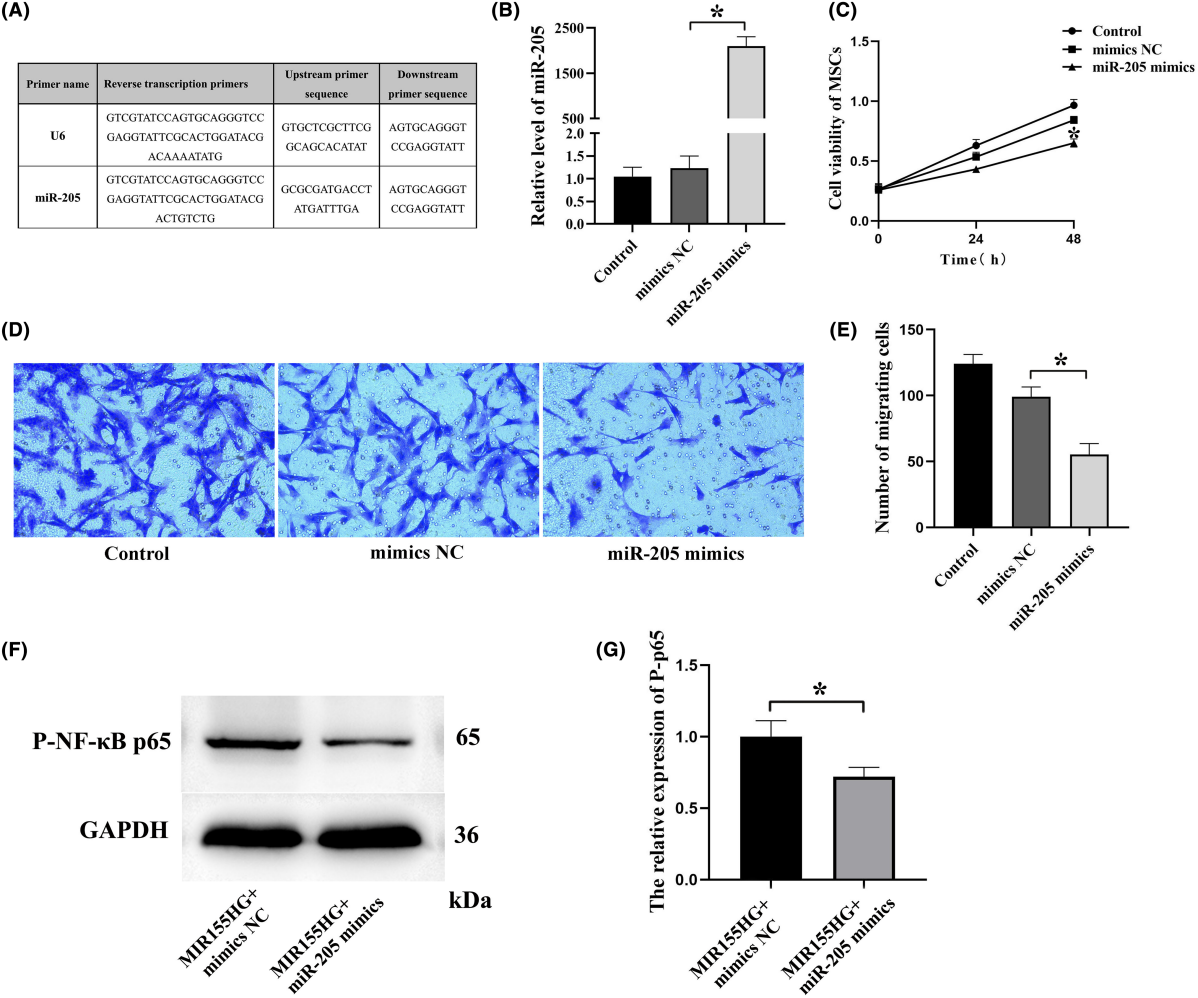
miR‐205 suppresses the viability and migration of MSCs. (A) Reverse transcription and primer sequence of miR‐205. (B) Transfection efficiency of miR‐205 in MSCs by qRT‐PCR, *p < 0.05. (C) Cell viability by CCK‐8 assay, *p < 0.05. (D) Representative images of transwell assay. (E) Migratory ability of MSCs after miR‐205 transfection, *p < 0.05. (F) Representative western blots of p‐NF‐κB p65 expression in MSCs. (G) Quantitative analysis of western blots, *p < 0.05.

In this part of the experiment, miR‐205 was successfully transfected into MSCs (Figure 6A,B). miR‐205 significantly reduced the viability of MSCs (Figure 6C). In addition, the results of the transwell assay showed that miR‐205 significantly reduced the migration ability of MSCs (Figure 6D,E). This conclusion might be consistent with that miR‐205 working as a suppressor. The expression of phosphorylated NF‐κB p65 in the MIR155HG + miR‐205 mimics group was significantly reduced than in the MIR155HG + mimics NC group (Figure 6F,G). This suggests that the NF‐κB pathway is involved in MIR155HG/miR‐205 interaction.

### 3.6 MIR155HG improves apoptosis and angiogenesis in vitro through sponging miR‐205

**FIGURE 7:**
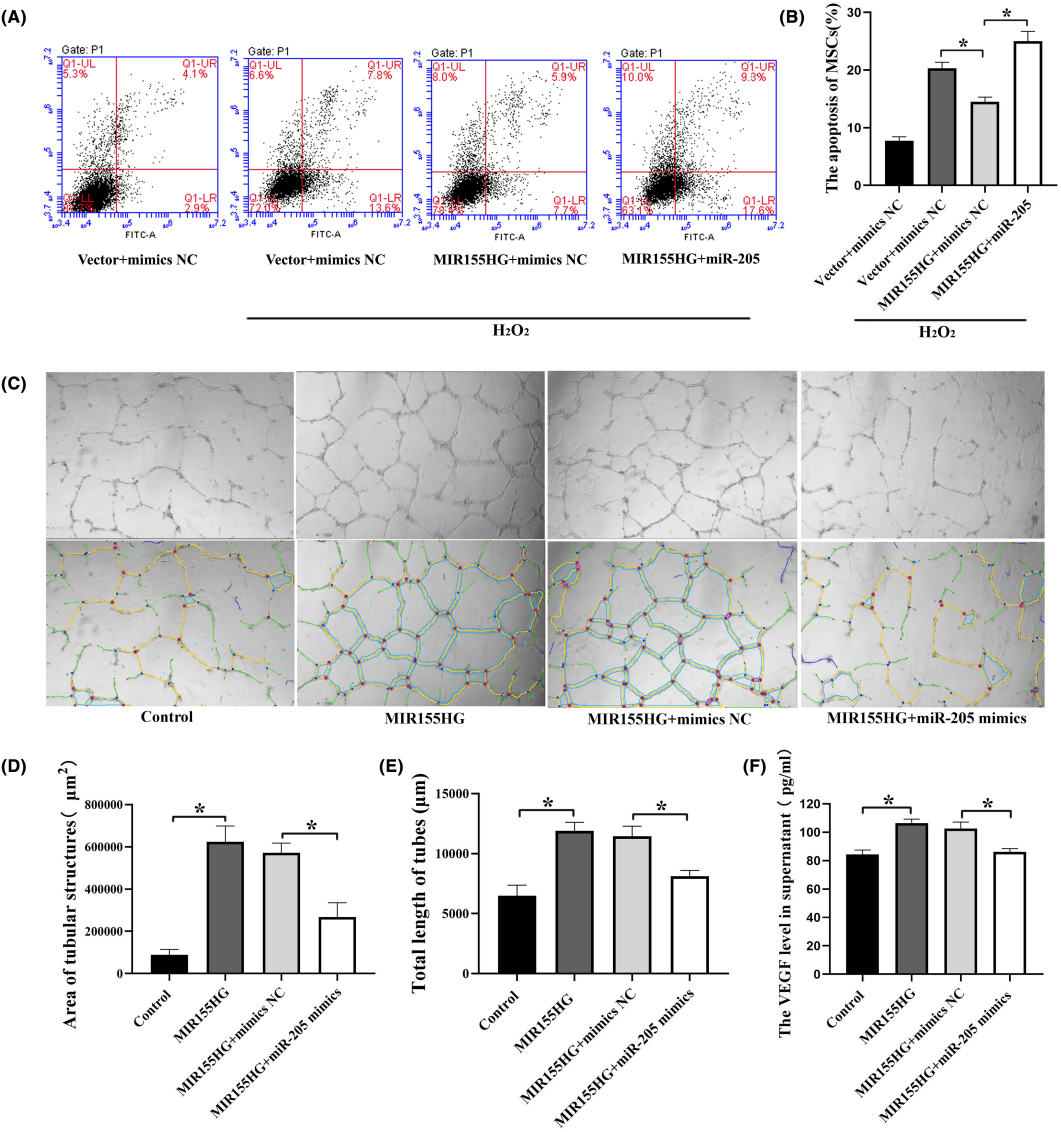
MIR155HG improves apoptosis and angiogenesis in vitro by sponging miR‐205. (A) Representative images of cell apoptosis detected by flow cytometry. (B) Analysis of the role of miR155HG/miR‐205 ceRNA on the apoptosis of MSCs, *p < 0.05. (C) Representative images of endothelial cell tube formation. The indicators of tube formation were analysed by Image J software. (D, E) Quantitative analysis of tubular forming ability of HUVECs, *p < 0.05. (F) Secretion of VEGF in the supernatant of MSCs detected by Elisa, *p < 0.05.

MIR155HG significantly reduced the apoptosis of MSCs under oxidative stress. However, the co‐transfection with miR‐205 reversed this protective effect (Figure 7A,B). These results suggested that MIR155HG alleviated the apoptosis of MSCs by sponging miR‐205. In addition, the culture supernatant from MIR155HG‐MSCs enhanced the tube formation in HUVECs. And the tube formation was inhibited in MIR155HG + miR‐205 mimics group compared with the MIR155HG+ mimics NC group (Figure 7C–E). The results showed that MIR155HG promoted angiogenesis in vitro by sponging miR‐205. As a kind of common pro‐angiogenesis factor, the VEGF level in the supernatant was significantly increased after MIR155HG overexpression, but the promoting effect of MIR155HG on VEGF was inhibited by miR‐205 mimics (Figure 7F). This result was consistent with the result of the tube formation experiment. MIR155HG exerted an endothelial promoting effect on MSCs by sponging miR‐205.

## 4 DISCUSSION

As the favourite source of stem cell therapy, MSCs possess multipotency.^23^ Several studies use MSCs to treat vascular IH.^24^, ^25^, ^26^ However, the role of MSCs in vascular injury remains to be fully elucidated, and the treatment effect of MSCs alone is not satisfactory. Therefore, in this work, lncRNA was used to improve the function of MSCs to enhance the therapeutic effect of MSCs. Some lncRNAs regulate VEC proliferation (e.g. MALAT1 and H19) or angiogenesis (e.g. MEG3 and MANTIS).^27^, ^28^ Several lncRNAs affect VSMC phenotypic transition or vascular remodelling (e.g. ANRIL, SMILR and SENCR).^29^, ^30^ At present, most studies on MIR155HG involve tumour or immunity, but few of them involve vascular remodelling. MIR155HG is transcribed from a gene located on chromosome 21q21 and consists of three exons that span 1.5 Kb in length.^31^, ^32^

In this study, lentivirus transfection was used to obtain MSCs with sustained high MIR155HG expression. MIR155HG significantly reduced the proportion of MSCs in the G1 phase and increased the proportion of those in the S phase. The results suggested that MIR155HG promoted the proliferation of MSCs. Furthermore, the migratory ability of MSCs was also significantly improved by MIR155HG. In addition, MIR155HG significantly increased the phosphorylation level of NF‐κB, while NF‐κB inhibitor decreased the phosphorylation of NF‐κB and inhibited the proliferation and migration of MSCs induced by MIR155HG. These results confirmed that the NF‐κB pathway was involved in the regulation of MIR155HG on the proliferation and migration of MSCs. Lin et al. also reported that MIR155HG overexpression increases the phosphorylation levels of NF‐κB p65 in gastric cancer cells.^19^ Some lncRNAs regulate the cell viability through increasing the transcription of pro‐inflammatory cytokines by enhancing inflammatory signals, such as NF‐κB signalling.^33^ The in vivo experiments revealed that the intimal thickness and collagen deposition were reduced in MIR155HG‐MSCs group. The results confirmed that MIR155HG improved the functions of MSCs resulting in a more effective relief in IH. This will provide a new therapeutic idea for prolonging the life span of the grafted vessels.

LncRNAs interact with specific miRNAs through miRNA response elements, which are sequences within secondary structures.^34^ This interaction results in the reduction of miRNA expression, decrease of miRNA activity and functional regulation of the corresponding genes. However, the effect of ceRNA regulation depends on the relative expression of lncRNAs and miRNAs. Hence, changes at the ceRNA level are critical for regulatory processes in different organs and tissues.^35^ Recent studies focused on the roles of ceRNAs in the tumorigenesis and development of various cancers. This lncRNA/miRNA cross‐talk modulates the gene expression driving pathological processes, such as angiogenesis and cell migration.^36^, ^37^ To some extent, we hold the opinion that these pathological processes are similar and related to some aspects of the cardiovascular diseases.^38^

Therefore, ceRNA mechanism in the regulation of MSCs by MIR155HG was investigated. Ten miRNAs significantly down‐regulated by MIR155HG were screened, and miR‐205 was selected for further study. In vitro experiments confirmed that miR‐205 reduced the cell activity and migration of MSCs, exerting the opposite effect of MIR155HG on MSCs. An effective binding sequence between MIR155HG and miR‐205 was verified. These results confirmed that MIR155HG acted as the ceRNA of miR‐205 in the regulation of MSCs.

The secret of successful cell therapy may lie, along with the homing, in the release of paracrine factors, such as immunomodulation factors, angiogenic factor, antiapoptotic factors and antioxidative factors.^39^, ^40^, ^41^ The anti‐apoptotic ability is essential for MSC therapy. MIR155HG/miR‐205 ceRNA reduced the apoptosis of MSCs under oxidative stress, suggesting that MSCs exerted a better therapeutic effect in the target tissue. Moreover, MIR155HG improved the angiogenesis of endothelial cells by down‐regulating miR‐205. This might be related to MIR155HG's promotion of VEGF secretion by MSCs. The VEGF level in the supernatant was significantly increased after MIR155HG overexpression, but the promoting effect of MIR155HG on VEGF was inhibited by miR‐205 mimics. MIR155HG/miR‐205 regulates the effect of MSCs on angiogenesis through paracrine. The improvement of endothelial cell function contributes to the repair of damaged blood vessels, alleviating the progress of IH.

## 5 CONCLUSIONS

In conclusion, lncRNAs and miRNAs are pivotal regulators in the multifunction of MSCs by modulating target gene expression or signalling pathway. MIR155HG/miR‐205 ceRNA contributes to improve the efficacy of MSCs in the treatment of IH. Indeed, ceRNA regulation of MIR155HG/miR‐205 to MSCS was elaborated, providing a new theoretical basis and a good application prospect for the intervention on IH and improvement of the surgical treatment of coronary heart disease, although further research on MIR155HG should be performed before clinical application.

## AUTHOR CONTRIBUTIONS

**Xiao Bai:** Conceptualization (lead); data curation (lead); formal analysis (equal); methodology (equal); writing – original draft (lead). **Zaiwen Qi:** Data curation (equal); formal analysis (equal); methodology (equal). **Chuanliang Cai:** Formal analysis (equal); supervision (equal). **Hao Song:** Formal analysis (equal); methodology (equal). **Guangmin Song:** Funding acquisition (equal); investigation (equal); supervision (equal). **Xin Zhao:** Funding acquisition (equal); investigation (equal); supervision (equal); writing – review and editing (lead).

## CONFLICT OF INTEREST STATEMENT

The authors confirm that there are no conflicts of interest.

## Data Availability

The data that support the study are available from the corresponding author upon reasonable request.
